# Impact of Hypertensive Disorders of Pregnancy on Stillbirth and Other Perinatal Outcomes: A Multi-Center Retrospective Study

**DOI:** 10.7759/cureus.22788

**Published:** 2022-03-03

**Authors:** Marina Basta, Kiran Hanif, Sana Zafar, Abdalla Khabazeh, Faiqa Amin, Sabeen Sharif Khan, Umar Ghaffar, Fares Mohammed Saeed Muthanna, Sher Wali

**Affiliations:** 1 School of Medicine, St. George's University, True Blue, GRD; 2 Medicine, Ayub Medical College, Abbottabad, PAK; 3 Biological Sciences, Victoria University of Wellington, School of Biological Sciences, Wellington, NZL; 4 Faculty of Medicine, Damascus University, Damascus, SYR; 5 Medicine, Quaid e Azam Medical College, Bahawalpur, PAK; 6 Medicine, Aga Khan University Hospital, Karachi, PAK; 7 Internal Medicine, Nishtar Medical University and Hospital, Multan, PAK; 8 Department of Pharmaceutical Care, Walailak University, Nakhon Si Thammarat, THA; 9 Research, Aga Khan University, Karachi, PAK

**Keywords:** impact, adverse perinatal outcomes, perinatal outcomes, stillbirth, hypertensive disorders of pregnancy

## Abstract

Objective: The aim of the study is to assess the impact of hypertensive disorders of pregnancy on stillbirths and other perinatal outcomes among women in Karachi, Pakistan.

Methodology: This was a retrospective cohort study conducted at two tertiary care hospitals, Aga Khan Hospital (AKU) and Liaquat National Hospital (LNH) in Karachi, Pakistan. The primary outcome variable of this study was stillbirth. Other outcomes assessed in this study included preterm birth, low birth weight, and early neonatal death.

Results: Data of a total of 840 women were included in this study; 280 (33.33%) women had hypertensive disorders of pregnancy and 560 (66.67%) were normotensive. Among women who had hypertensive disorders of pregnancy, the adjusted odds ratio (AOR) of having a stillbirth was two times more than that for normotensive women (AOR=2.62, 95% CI=1.46-4.40), four times for low birth weight (AOR=4.23, 95% CI=2.88-6.20), five times for early neonatal death (AOR=5.03, 95% CI=2.40-10.50) and six times for pre-term birth (AOR=5.16, 95% CI=3.42-7.79).

Conclusion: The current study found that incidence of stillbirth, low birth weight, pre-term birth, and neonatal mortality is higher in mothers with hypertensive disorders of pregnancy than normotensive mothers.

## Introduction

Stillbirths are a major global issue that has gotten little attention. Each year, an estimated 2.6 million babies are stillborn, with 98% of them taking place in low and middle-income nations [1]. Efforts have been made to recognize the causes of stillbirth. For instance, stillbirths are known to be linked to pregnancy complications, and hypertensive disorders of pregnancy (HDP) are the most common cause of pregnancy complications [2]. Disorders such as gestational hypertension, pre-eclampsia or eclampsia, superimposed preeclampsia and chronic hypertension occur in 3-8% of pregnancies all over the world [3]. These four subgroups are thought to play various roles in stillbirth due to their diverse pathogenic processes and clinical symptoms [4]. Also, they are an important cause of perinatal and maternal morbidity [5].

HDP is associated with endothelial damage, oxidative stress, and vascular manifestations. This affects placental function due to poor nutrient supplementation and perfusion to the fetus leading to adverse perinatal outcomes [6].

Hypertensive disorders of pregnancy account for 15% of perinatal deaths worldwide [7]. Around 16% of the estimated 2.6 million stillbirths each year occur in pregnancies affected by HDP [8]. Eight neonatal deaths out of 1000 live births are caused by hypertension disease of pregnancy [9]. The survey conducted by World Health Organization (WHO) found that pre-eclampsia and eclampsia were the major obstetrical cause of 25% of perinatal deaths with similar proportions affected by early neonatal deaths and stillbirths [10].

Few large-scale studies have looked into the link between hypertensive conditions and stillbirth during pregnancy but most of these studies have been done in high-income countries. There are very few studies related to the impact of HDP on perinatal outcomes in Pakistan. The incidence of perinatal mortality is high in Pakistan and HDP can be postulated to be one of the reasons for the higher incidence of mortality and morbidity like low birth weight, pre-term birth, fetal death, and perinatal outcomes. Therefore, the purpose of this study is to determine the impact of hypertensive disorders of pregnancy on stillbirth and other perinatal outcomes among women in Karachi, Pakistan.

## Materials and methods

We performed a retrospective cohort study at two tertiary care hospitals, namely Aga Khan Hospital (AKU) and Liaquat National Hospital (LNH) in Karachi, Pakistan. The data for this study were retrieved through the hospital management information system (HMIS). Women who gave birth at AKU or LNH from January 2019 to December 2021 were included in the study. Women with missing data including gestational age at birth, birth weight, and age of newborn at death were excluded from the study. Besides this, more than singleton births and mothers with Rh sensitization, diabetes mellitus, gestational diabetes, and incompetent cervix were also excluded from the study based on patient electronic medical records (EMR) from HMIS.

For the sample size, we took a ratio of exposed to the unexposed group as 1:2. Firstly, we identified women with hypertensive disorder of pregnancy (exposed group) using EMR of both hospitals. Then, we identified 278 women with HDP who fulfilled eligibility criteria and included them in the final analysis. To identify the unexposed group, a list of all eligible normotensive women who gave birth at AKU or LNH during the same duration as the exposed group was obtained from the EMR. Simple random sampling using a computer random number generator was done to select women of the unexposed group. Data of a total of 556 normotensive women were included in the study. We selected 150 and 128 exposed women from AKU and LNH respectively, while 278 unexposed women were selected from both of the above-mentioned hospitals. 

Outcome variables

The primary outcome variable of this study was stillbirth, which is defined as death at or after 28 weeks of gestation. Other outcomes assessed in this study included preterm birth (birth before 37 weeks of gestation), low birth weight (less than 2500 g), and early neonatal death (death that occurred in the first seven days of delivery) [11]. 

The primary exposure variable is hypertensive disorders of pregnancy. According to American College of Obstetricians and Gynecologists guidelines, HDP are classified into four categories including gestational hypertension, preeclampsia or eclampsia, superimposed preeclampsia, and chronic hypertension [12]. Chronic hypertension was defined as hypertension before pregnancy or before 20 weeks of gestation. Superimposed preeclampsia was defined as chronic hypertension associated with preeclampsia. After 20 weeks of pregnancy, preeclampsia was defined as hypertension with proteinuria, or hypertension along with the involvement of at least one organ system. When preeclampsia advanced to the convulsive stage, eclampsia was diagnosed. Gestational hypertension was defined as new-onset hypertension after 20 weeks of pregnancy, with blood pressure returning to normal by 12 weeks after delivery [2]. Other variables included in the study were the age of mother, parity, delivery mode, gestational age in weeks, fetus gender, anemia in mothers, number of antenatal care visits, body mass index (BMI), previous history of stillbirth and fetal malpresentation at birth, and other comorbidities in mothers. Data were gathered from HMIS using a pre-designed questionnaire.

Statistical analysis

Data were analyzed using STATA version 16.0. Continuous data were presented as mean and standard deviation, while categorical variables were presented as frequency and percentages. Comparative analysis between normotensive women and women with HDP was done using T-tests and chi-square tests for continuous and categorical variables respectively. All significant thresholds were set at a p-value<0.05. Multivariable logistic regression was used to determine the relationship of hypertensive disorders of pregnancy with outcomes including stillbirth, preterm birth, low birth weight, and early neonatal death while adjusting for confounding variables. Adjusted odds ratios (AOR) were presented along with a 95% confidence interval.

## Results

Data of a total of 834 women were included in this study; 280 (33.33%) women had hypertensive disorders of pregnancy and 560 (66.67%) were normotensive. Among 280 women with hypertensive disorders of pregnancy, 6.47% had chronic hypertension, 10.43% had gestational hypertension and 65.83% and 17.27% had preeclampsia and eclampsia respectively.

Table 1 shows the comparison of characteristics between normotensive mothers and mothers with hypertensive disorders of pregnancy. The mean age of normotensive mothers and mothers with hypertensive disorders of pregnancy was 27.01 +/- 4.88 and 26.25 +/- 5.27 years and it was significantly different between the two groups (p-value=0.041). The frequency of primary gravida was significantly higher in mothers with hypertensive disorders of pregnancy than normotensive mothers (42.85% vs. 32.86%) (p-value=0.006). The proportion of normal BMI was significantly higher in normotensive mothers than mothers with hypertensive disorders of pregnancy (60.36% vs. 50.71%). Other characteristics that were significantly different between normotensive mothers and mothers with hypertensive disorders of pregnancy included antenatal care visits (p-value=0.001), fetal malpresentation (p-value=0.001), mode of delivery (p-value=0.001), and previous history of stillbirth (p-value=0.001).

**Table 1:** Comparison of characteristics of normotensive mothers and mothers with hypertensive disorders of pregnancy * Significant at p-value<0.05 ^ Mean (Standard deviation) BMI: Body mass index

Variables	Categories	Hypertensive Disorders of Pregnancy		P-value
		No n(%)	Yes n(%)	
Age^		27.01 +/- 4.88	26.25 +/- 5.27	0.041*
Gravida	Primary gravida	184 (32.86)	120 (42.85)	0.006*
	Multipara	293 (52.32)	115 (41.07)	
	Grand multipara	83 (14.82)	45 (16.07)	
BMI	Underweight	29 (5.18)	23 (8.21)	0.022*
	Normal	338 (60.36)	142 (50.71)	
	Overweight	105 (18.75)	68 (24.28)	
	Obesity	88 (15.71)	47 (16.78)	
Anemia at the time of delivery	No	108 (19.29)	64 (22.85)	0.307
	Yes	452 (80.71)	216 (77.14)	
Antepartum Hemorrhage	No	492 (87.86)	238 (85.00)	0.291
	Yes	68 (12.14)	42 (15.00)	
Antenatal care visit	0	13 (2.32)	13 (4.64)	0.001*
	1 to 3	211 (37.68)	147 (52.50)	
	4 or more	336 (60.00)	120 (42.86)	
Fetal Malpresentation	No	422 (75.36)	246 (87.86)	0.001*
	Yes	138 (24.64)	34 (12.14)	
Mode of Delivery	Vaginal	238 (42.50)	174 (62.14)	0.001*
	Cesarean section	322 (57.50)	106 (37.86)	
Past history of stillbirth	No	551 (98.39)	240 (85.71)	0.001*
	Yes	9 (1.61)	40 (14.29)	
Previous history of eclampsia or pre-eclampsia	No	488 (87.14)	215 (76.78)	0.001*
	Yes	72 (12.58)	65 (23.21)	

Table 2 shows the incidence of stillbirth, low birth weight, early neonatal death, and pre-term birth among study participants. The incidence of stillbirth was significantly higher in mothers with HDP than normotensive mothers (13.67% vs. 5.00%). Women with HDP are more likely to have a child with low birth weight (40.71%) than normotensive mothers (12.50%). Pre-term births among mothers with HDP and normotensive mothers were about 40.36% and 10.36% respectively and it was significantly different (p-value=0.001). Lastly, the incidence of early neonatal death was significantly higher in mothers with HDP than in normotensive mothers (p-value=0.001).

**Table 2:** Univariate analysis of comparison of outcome between normotensive mothers and mothers with hypertensive disorders of pregnancy * Significant at p-value<0.05

Outcome	Categories	Hypertensive Pregnancy Disorders		P-value
		No n(%)	Yes n(%)	
Stillbirth	No	532 (95.00)	241 (86.33)	0.001*
	Yes	28 (5.00)	39 (13.67)	
Low birth weight	No	490 (87.50)	166 (59.28)	0.001*
	Yes	70 (12.50)	114 (40.71)	
Early Neonatal death	No	519 (97.56)	249 (88.93)	0.001*
	Yes	13 (2.44)	31 (11.07)	
Pre-term Birth	No	502 (89.64)	167 (59.64)	0.001*
	Yes	58 (10.36)	113 (40.36)	

Table 3 shows the impact of hypertensive disorders of pregnancy on prenatal outcomes after adjusting variables such as age, gender, gravida, BMI, anemia at the time of delivery, antepartum hemorrhage, antenatal care visits, fetal malpresentation, mode of delivery, past history of stillbirth and previous history of eclampsia and preeclampsia. Among women with HDP, the adjusted odds ratio of having stillbirth was more than two times that for normotensive women (AOR=2.62, 95% CI=1.46-4.40), four times for low birth weight (AOR=4.23, 95% CI=2.88-6.20), five times for early neonatal death (AOR=5.03, 95% CI=2.40-10.50) and six times for pre-term birth (AOR=5.16, 95% CI=3.42-7.79). Among the four types of hypertensive disorders of pregnancy, eclampsia was associated with the greatest odds of adverse events with five times the odds of having a stillbirth (AOR=5.16, 95% CI=1.42-18.68), more than seven times the odds for pre-term birth (AOR=7.44, 95% CI=3.67-15.08) and early neonatal death (AOR=16.19, 95% CI=3.33-37.69). Chronic hypertension was associated with the greatest odds of low birth weight with six times the odds of having low birth weight (AOR=6.19, 95% CI=2.24-17.13) as shown in Table 3.

**Table 3:** Multivariate analysis on the impact of all forms of HDP on perinatal outcomes * Significant at p-value<0.05 AOR: Adjusted odds ratio

Outcomes	AOR	95% CI	P-value
	Impact of all Hypertensive Pregnancy Disorders		
Stillbirth	2.62	1.46-7.40	0.001*
Low birth weight	4.23	2.88-6.20	0.001*
Early Neonatal death	5.03	2.40-10.50	0.001*
Pre-term Birth	5.16	3.42-7.79	0.001*
	Impact of Chronic Hypertension		
Stillbirth	3.03	1.14-8.60	0.026*
Low birth weight	6.19	2.24-17.13	0.001*
Early Neonatal death	12.66	4.12-38.78	0.001*
Pre-term Birth	6.25	2.19-17.82	0.001*
	Impact of Gestational Hypertension		
Stillbirth	1.39	0.29-6.57	0.676
Low birth weight	0.98	0.32-3.03	0.974
Early Neonatal death	1.47	0.18-12.29	0.718
Pre-term Birth	1.54	0.52-4.58	0.432
	Impact of Preeclampsia		
Stillbirth	2.66	1.42-4.97	0.002*
Low birth weight	5.45	3.56-8.37	0.001*
Early Neonatal death	4.77	2.08-10.87	0.001*
Pre-term Birth	5.71	3.60-9.02	0.001*
	Impact of Eclampsia		
Stillbirth	5.16	1.42-18.68	0.012*
Low birth weight	4.46	2.24-8.87	0.001*
Early Neonatal death	16.19	3.33-37.69	0.001*
Pre-term Birth	7.44	3.67-15.08	0.001*

## Discussion

This paper compared the magnitude of the risks of perinatal outcomes including stillbirth, low birth weight, pre-term birth and early neonatal mortality between normotensive mothers and mothers with HDP. The study found that hypertensive disorders of pregnancy have a significant impact on stillbirth, low birth weight, pre-term birth and early neonatal mortality after adjusting for potential confounders. These findings were reaffirmed in a previous study conducted by Allen et al. who reported adverse perinatal outcomes associated with HDP, such as low birth weight, stillbirth, and small gestational age [13].

Globally, HDP is estimated to occur in 5% to 10% of pregnancies [14]. Developing countries are known to be disproportionally affected by HDP. Previous studies conducted in low- and middle-income countries found that the estimated prevalence of eclampsia and preeclampsia is 7% to 18% [14-15]. Our study established an overall prevalence of hypertensive disorders of pregnancy.

The incidence of stillbirth among women with HDP was 13.67%. The findings of our study were consistent with studies conducted by Rush (10%) [16] and Seck and Jackson (10.2%) [17]. In some studies, the incidence of stillbirth among women with HDP is quite higher, e.g. 23.5% in Hawassa, Ethiopia [18] and 22.1% in Bicol, Philippines [19]. This disparity could be related to differences in the quality of prenatal care and obstetrics treatment provided by different health organizations, as well as the study design used. However, the findings of all these studies have shown that the risk of stillbirth is higher among women with HDP as compared to normotensive women which might be related to the effect of placental ischemia and maternal mal-perfusion linked to HDP [2].

The current study found that the risk of delivering pre-term babies among mothers with HDP is higher than normotensive mothers, which was accordant with the study conducted by Jaleta et al. [20]. This could be owing to the fact that interventional deliveries are performed regardless of gestational weeks. Early delivery is carried out in women with severe preeclampsia and eclampsia subtypes of hypertensive disorders of pregnancy to avoid further unfavorable maternal and neonatal outcomes. Premature births are one of the leading causes of early neonatal deaths [21]. Therefore, managing and preventing hypertensive disorders of pregnancy should be an important aspect to prevent neonatal deaths and accelerate the progress towards the accomplishment of enhancing neonatal survival in Pakistan.

Our study also found that the incidence of low birth weight is higher in mothers with HDP as compared to normotensive mothers. This was reiterated in a study conducted by Siabani et al. [22], who also found that the mean birth weight is significantly higher in normotensive mothers as compared to the mothers with HDP. This could be explained by vascular manifestation disruption seen in HDP, which affects placental function, resulting in poor fetal perfusion and nutrient supply. As a result, lowering the risk of low birth weight necessitates extra attention to immediate post-natal care that includes keeping the newborn warm, initiating skin-to-skin contact, as well as aiding with early breastfeeding.

In the present study, a low number of antenatal visits were reported in women with HDP as compared to normotensive women which is associated with enhanced risk of stillbirth and other serious perinatal outcomes. Thus, timely provision of high-quality antenatal care is vital along with the empowerment of women through the provision of health-related information to decrease adverse health outcomes.

In order to enhance perinatal outcomes, health care practitioners should focus on hypertensive disorders of pregnancy prevention, early diagnosis, and fast treatment. Interventions aimed at reducing newborn mortality, as well as other predictors of perinatal death, will help to accomplish global and national sustainable development goals.

This is one of few studies conducted in Pakistan that determined the impact of hypertensive disorders of pregnancy on stillbirth and other perinatal outcomes. The study included the data of two tertiary care hospitals in Karachi. The current study has certain limitations. Firstly, it was a retrospective study, therefore we were unable to assess certain confounding factors such as smoking status, nutritional status, educational status, and environmental factors. Secondly, there could be differences in the quality of prenatal care and obstetrics treatment provided by different hospitals affecting the adverse outcomes.

Thus, prospective studies need to be conducted in the near future that considers different factors including sociodemographic characteristics and environmental factors in Pakistan.

## Conclusions

The current study found that incidence of stillbirth, low birth weight, pre-term birth, and neonatal mortality is higher in mothers with hypertensive disorders of pregnancy than normotensive mothers. In particular, women with eclampsia are susceptible to the most severe form of hypertensive disorder of pregnancy which has the worst health outcomes.
